# S1-induced vasospastic angina—diagnostic utility of Holter ECG: a report of a case

**DOI:** 10.1186/s40792-020-00975-x

**Published:** 2020-08-24

**Authors:** Tatsuo Kanda, Atsuhiro Wakai, Tadasu Chida, Yuichi Nakamura

**Affiliations:** 1 Department of Surgery, Sanjo General Hospital, Tsukanome, Sanjo, Niigata, 955-0055 Japan; 2 Department of Cardiology, Nagaoka Chuo General Hospital, Nagaoka, Japan

**Keywords:** Chemotherapy, Gastric cancer, Holter ECG, S1, Vasospastic angina

## Abstract

**Background:**

Vasospastic angina is a rare but potentially life-threatening adverse event (AE) of S1, an oral fluoropyrimidine anticancer agent. However, this AE is not well known owing to its low incidence. We report herein a case of a patient who suffered from vasospastic angina associated with S1 chemotherapy for unresectable gastric adenocarcinoma, along with a review of the literature.

**Case presentation:**

A 68-year-old woman was endoscopically diagnosed with gastric adenocarcinoma of the diffuse type. Abdominal pelvic contrast-enhanced computed tomography (CT) revealed small nodules in the omentum and ascites in the pouch of Douglas. The patient was clinically diagnosed with unresectable gastric adenocarcinoma with peritoneal metastasis, and primary chemotherapy with S1 plus cisplatin was selected. Around midnight of day 1, the patient complained of sudden oppressive chest pain. The pain disappeared spontaneously after 3–5 min, but similar events happened every midnight thereafter. No significant change was found on bedside electrocardiograms (ECGs) recorded immediately after the pain attacks. The patient was suspected to have unstable angina and underwent Holter ECG on day 4 of treatment. Holter ECG revealed ST segment elevations and short-run ventricular tachycardia during a pain attack. S1 chemotherapy was discontinued, and no attack was observed thereafter. Coronary CT angiography showed no significant stenosis of coronary arteries.

**Conclusions:**

Clinicians should be aware of vasospastic angina as a serious AE in the chemotherapy with S1. Holter ECG is useful for the early diagnosis of this rare and clinically important AE.

## Background

S1, an oral fluoropyrimidine anticancer agent, is widely used for the treatment of unresectable and/or metastatic gastric cancer and for adjuvant chemotherapy after potentially curable resection of stage II/III gastric cancer in Japan [1]. Vasospastic angina is a rare but potentially life-threatening adverse event (AE) of 5-fluorouracil (5-FU) [2] and other orally available prodrugs [3], including S1. This AE is, however, not well known owing to its low incidence. We report herein a case of a patient who suffered from vasospastic angina associated with S1 chemotherapy for unresectable gastric adenocarcinoma, along with a review of the literature.

## Case presentation

A 68-year-old woman visited our hospital, complaining of epigastralgia. The patient underwent gastroduodenal endoscopy and was diagnosed with gastric adenocarcinoma of the diffuse type. Abdominal pelvic contrast-enhanced computed tomography (CT) revealed small nodules in the omentum and ascites in the pouch of Douglas. The patient was clinically diagnosed with unresectable gastric adenocarcinoma with peritoneal metastasis. Primary chemotherapy with S1 plus cisplatin was planned, and the patient was hospitalized for the introduction of the chemotherapy. The patient has no history of cardiac diseases or surgery. Despite being a smoker (Brinkman Index, 480), she has no other risks of ischemic heart diseases, including hypertension, hyperlipidemia, or hyperuricemia. Her height and weight were 143 cm and 35.8 kg, respectively. Electrocardiography (ECG) on admission showed a normal sinus rhythm.

**Fig. 1:**
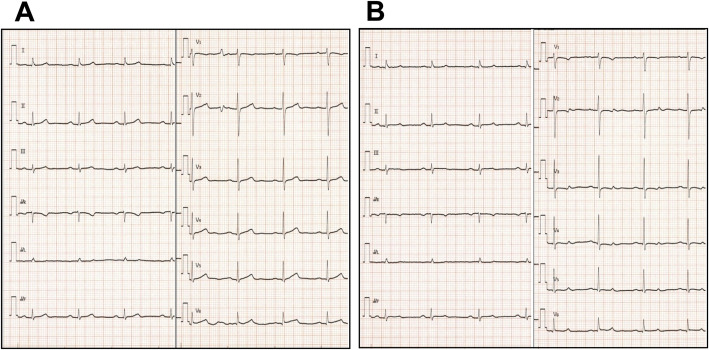
Electrocardiograms (ECGs) recorded on bedside. The patient complained of angina after sleep (10 PM) and midnight (1 AM) on day 2 as well as day 1 of S1 chemotherapy. Bedside ECGs were incapable of capturing abnormal findings although they were conducted immediately after the attacks. **a** After sleep. **b** Midnight

**Fig. 2:**
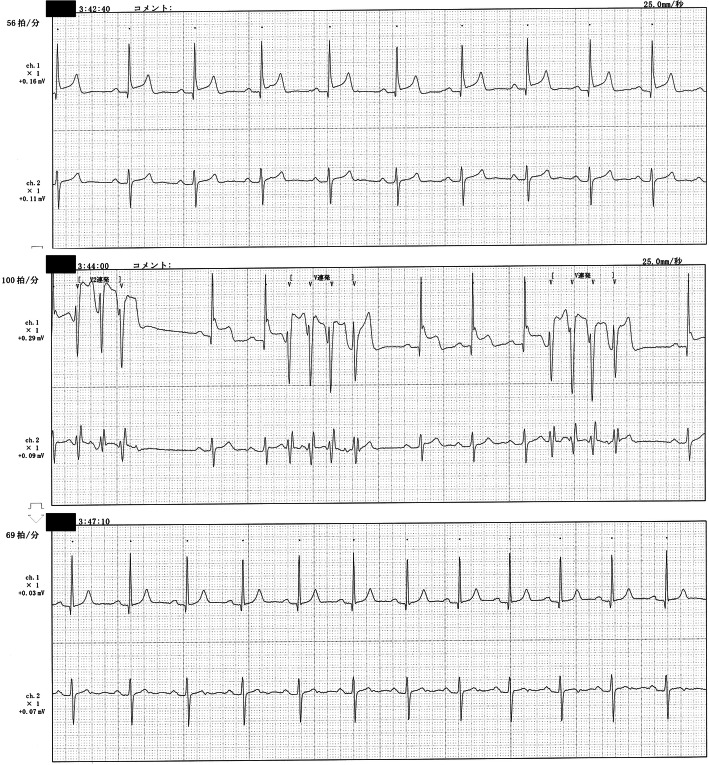
Holter electrocardiogram (ECG). The patient’s ECG was continuously monitored on day 4 of treatment. Holter ECG revealed that ST segment elevations appeared together with angina (upper panel) and were followed by short-run tachycardia 1 min later (middle panel). The abnormal findings on ECG disappeared 4 min after sublingual nitroglycerin administration (lower panel)

**Fig. 3:**
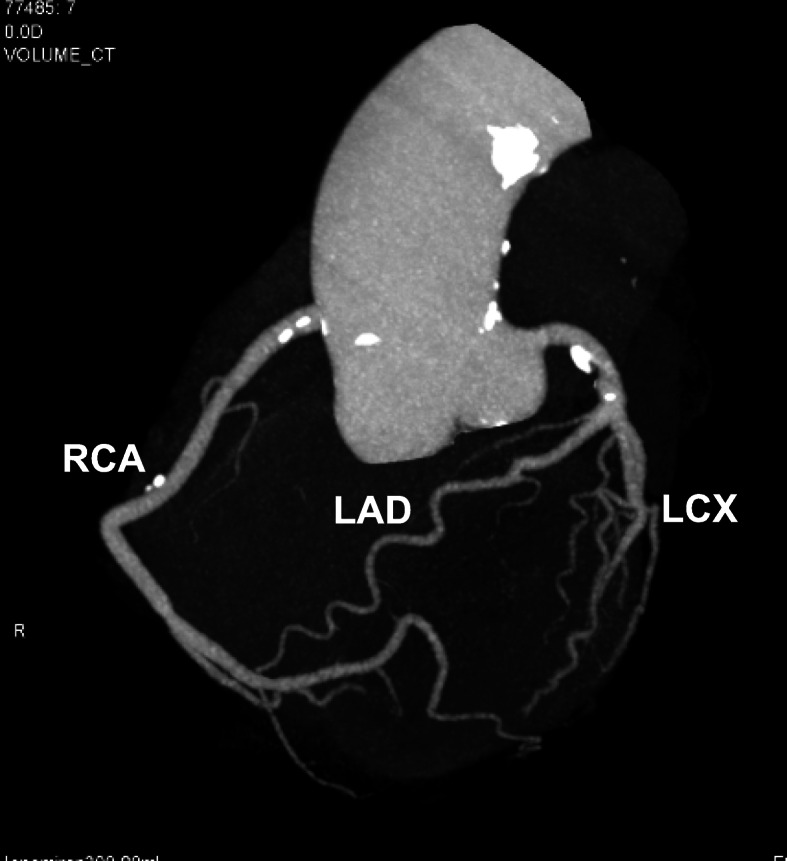
Coronary computed tomography (CT) angiography. Coronary CT angiography revealed no significant stenosis of coronary arteries although dispersed small spotty calcifications were noted. LCX, left circumflex artery; LAD, left anterior descending artery; RCA, right coronary artery

The patient started S1 administration at the dosage of 80 mg daily. Around midnight of day 1 of the treatment, the patient complained of sudden oppressive chest pain. The pain disappeared spontaneously after 3–5 min, but similar events happened every midnight thereafter. No significant change was recorded on bedside ECGs measured immediately after the pain attacks (Fig. 1). The patient was suspected to have unstable angina because the pain was swiftly relieved after sublingual nitroglycerin administration, and underwent Holter ECG on day 4 of the treatment. The Holter ECG revealed ST segment elevations and short-run ventricular tachycardia during a pain attack (Fig. 2). No attack was observed after S1 administration was discontinued. Coronary CT angiography showed no significant stenosis of coronary arteries (Fig. 3). The chemotherapeutic regimen was changed to weekly paclitaxel plus ramucirumab. The patient continued the treatment for 10 months without cardiac AEs.

## Discussion

We present herein a case of vasospastic angina induced by S1 chemotherapy. Vasospastic angina has been known as a cardiac AE of 5-FU since the early 1970s [4]. S1, an orally available anticancer agent, also has a risk of cardiac AE because it is a 5-FU prodrug, containing tegafur and chemical modulators for its metabolism. The Pharmaceuticals and Medical Devices Agency (PMDA) of Japan reported angina and myocardial infarction as serious AEs (SAEs) of S1 in 2010. However, those SAEs are not yet widely known probably because of their low incidence.

To know the characteristics of S1-induced vasospastic angina, we searched the literature using PubMed database, designating “angina,” “cancer,” and “S1” as key words. Although we got three hits, careful examination revealed that only the case report of Shitara et al. [5] met our research purpose. Furthermore, we got nine hits after searching the Japana Centra Revuo Medicina, but found no suitable case report other than that of Shitara et al.

In that case report, a 58-year-old male patient with gastric cancer underwent S1 chemotherapy as an adjuvant treatment after gastrectomy. The patient had an angina attack 50 min after the first administration of S1. Coronary angiography conducted after discontinuation of S1 chemotherapy showed that the patient had no significant stenosis of coronary arteries. Notably, the cardiac AE of the patient occurred on day 1 of the treatment, as was observed in our case. As regards the timing of onset, a literature review indicated that cardiac AEs of 5-FU and related drugs tended to happen in the introduction period of the treatment [6–8]. Jensen et al. [9], who conducted a review of 668 patients with gastrointestinal malignancies who underwent chemotherapy with 5-FU or capecitabine in their institution, found 29 patients who showed cardiac AEs of severity grades 2–4 based on the Common Terminology Criteria for Adverse Events and characterized the patients on the basis of clinical background and treatment course. The cardiac AEs of 16 patients (55%) were diagnosed in the first course, with the median onset of treatment day 4. Their study revealed that more than half of these SAEs occurred in the initial stage of treatment and suggested that special attention should be given to the early phase of chemotherapy with 5-FU or related drugs including S1.

Although it remains unsettled, coronary vasospasm is assumed to be the most likely mechanism involved in cardiotoxicity brought about by 5-FU chemotherapy [2, 10] because angina happens suddenly at rest and subsides with nitroglycerin administration, and in some cases, calcium-channel inhibitors can prevent angina attack [9]. In our case as well, sublingual nitroglycerin swiftly relieved chest pain, and the patient’s ECG returned to normal immediately after the administration.

On the contrary, several studies using animal models have provided experimental evidence suggesting that 5-FU and its metabolites directly exert adverse effects on the myocardia. Matsubara et al. [11], using guinea pig experimental models, showed that the depletion of high-energy phosphate compounds and the accumulation of tricarboxylic acid cycle intermediates occurred in the ventricular myocardia in 5-FU-induced cardiotoxicity although myocardial blood flow remained constant. Mizuno et al. [12] reported a case of a 73-year-old female patient with 5-FU-induced cardiotoxicity. In that case, left ventricular angiography conducted in the prolonged cardiac attack with ST segment elevations revealed that the ventricular walls showed significant akinesis despite normal coronary angiographic findings. It may be also presumable that metabolic injuries of the myocardium underlie the mechanism of 5-FU-induced cardiomyopathy at least in some cases.

Despite no precise data on the incidence of 5-FU- or S1-induced cardiotoxicity, PMDA has classified the morbidity into SAE with unknown incidence of less than 0.5%. Rezkalla et al. [13] reported an intriguing clinical study on 5-FU-induced cardiotoxicity. In that study, the ECGs of 25 patients undergoing 5-FU chemotherapy were continuously monitored before and during the treatment using Holter ECG. Only one patient complained of angina, whereas 17 patients (68%) showed significant ST segment changes during 5-FU infusion. These findings suggest that subclinical cardiotoxicity may occur in patients receiving 5-FU chemotherapy more frequently than expected. S1 chemotherapy is generally initiated on an outpatient basis because the drug is orally available. Taking such clinical circumstances into consideration, there may be a tendency to overlook a significant number of patients with cardiac AEs in S1 chemotherapy.

The identification of risk factors would be useful to decrease the risk of this potentially lethal SAE. Jensen et al. addressed this in the above-mentioned study [9]. Unfortunately, they failed to determine the statistically significant risk factors for cardiac AEs although they found that the cardiac AEs of 5-FU occurred more frequently in patients with pre-existing cardiovascular diseases or low renal function than in those without. As regards S1 chemotherapy, there are only two case reports on S1-induced vasospastic angina, including the present case. So far, the best way for the early diagnosis of morbidity is to carefully record patient’s complaints particularly in the initial stage of S1 chemotherapy.

## Conclusions

We have presented a case of vasospastic angina associated with S1 chemotherapy. In the present case, although the patient suffered from the attacks around midnight, Holter ECG, which is able to detect abnormalities during the attack, could provide direct evidence for diagnosis. Clinical and surgical oncologists should be aware of this cardiac AE of S1 and have to take patient’s complaints seriously in the initial stage of treatment. Holter ECG is useful for diagnosis and recommended for suspected cases of this morbidity.

## Data Availability

Data supporting the conclusions are included in the article.

## Abbreviations

AE: Adverse event
CT: Computed tomography
ECG: Electrocardiography
5-FU: 5-Fluorouracil
PMDA: The Pharmaceuticals and Medical Devices Agency
SAE: Serious AE
